# Editorial: Educating the educators in digital STEM-education - the impact of teacher training and their further education

**DOI:** 10.3389/fpsyg.2026.1844824

**Published:** 2026-04-28

**Authors:** Melanie Basten, Kris-Stephen Besa, Dina Elhendawy, Daniela Fiedler, Jörg Großschedl, Nadine Großmann, Florian H. Müller, Andrea Maria Schmid, Matthias Wilde

**Affiliations:** 1 Department of Biology Education, Bielefeld University, Bielefeld, Germany; 2 General Didactics and Classroom Research, University of Münster, Münster, Germany; 3 Department of Science Education, University of Copenhagen, Copenhagen, Denmark; 4 Institute of Biology Education, University of Cologne, Cologne, Germany; 5 Faculty of Arts, Humanities and Education, University of Klagenfurt, Klagenfurt, Austria; 6 Institute of STEM and Sustainability Education, Lucerne University of Teacher Education, Lucerne, Switzerland

**Keywords:** digital STEM education, impact of teacher training, in-service and pre-service teacher training, teacher education, teacher professional development, Technological Pedagogical Content Knowledge (TPACK), technology integration in education

Effective digital Science, Technology, Engineering, and Mathematics (STEM) education is essential for addressing current global challenges, such as sustainability (Pérez-Martínez et al., 2023). In order to prepare future generations to thrive in an increasingly digital and scientifically advanced world, it is crucial to equip teachers with the necessary digital pedagogical skills (Organisation for Economic Co-operation and Development, 2025). Ongoing teacher education and professional development play a key role in building a scientifically literate, solution-oriented global society and are fundamental to the quality of formal STEM education (Huang et al., 2022). Digital methods can help bridge transitions between in-person and distance learning, support practical phases of instruction, and enhance both motivational and cognitive activation. Despite this potential, research on the effective use of digital media in STEM education remains fragmented. This interdisciplinary Research Topic (which is featured in both journals, Frontiers in Psychology and Frontiers in Education) brings together contributions that address these gaps by exploring how digital and hybrid learning environments shape teaching and learning in STEM disciplines. The articles span formal and non-formal settings and investigate a variety of digital tools, instructional approaches, and theoretical frameworks. A central theme emerging from the contributions to this Research Topic is the professional development of teachers through structured training formats, particularly in digitally enriched and out-of-school learning environments. These studies can be organized into three interconnected perspectives on teacher professional development in digital STEM education: (1) structured professional development programs, (2) the development of teacher competencies, beliefs, and knowledge, and (3) systemic and collaborative structures that support teacher learning. The articles included in this Research Topic address motivational, cognitive, technological, and pedagogical dimensions across different learning environments. Below, we summarize the key contributions of the included studies along these three perspectives.

(1) A central focus of this Research Topic lies in the design and evaluation of structured professional development programs that support teachers in integrating digital tools into STEM education. In this Research Topic, Reher et al. investigate in-service teacher training conducted in collaboration with out-of-school student laboratories, demonstrating improvements in teachers' professional knowledge, technological commitment, and self-efficacy, particularly in relation to domain-specific digital media. Similarly, Lüsse et al. analyze professional development programs in student laboratories and identify key success factors, such as hands-on activities, reflection, and collaborative learning opportunities. Digel and Roth extend this perspective by examining a structured professional development program that enhances students' conceptual understanding through technology-enhanced teaching, emphasizing the importance of transfer processes from teacher training to classroom practice. Finally, Offermann et al. focus on an online training format, showing how digital training can strengthen pre-service teachers' intentions, self-efficacy, and technological pedagogical and content knowledge for implementing digital scaffolds.

(2) A second group of contributions focuses on the development of teachers' competencies, beliefs, and professional knowledge related to digital STEM education. Abiltayeva et al. examine pre-service biology teachers' Technological Pedagogical Content Knowledge (TPACK), highlighting a gap between positive attitudes and actual implementation. Küng and Brovelli investigate how teacher training influences the evaluation and selection of augmented reality applications, emphasizing the role of knowledge-based decision-making. Hermann et al. explore teachers' professional vision when selecting and embedding explainer videos, revealing limitations in applying topic-specific pedagogical knowledge. Besa et al. further demonstrate the relationship between participation in professional development, self-efficacy, and TPACK, highlighting the importance of training for the motivational and cognitive aspects of teacher competence.

(3) Beyond individual training and competencies, two contributions emphasize the importance of systemic and collaborative structures that support teacher professional development. Biehl and Koerber analyze the implementation of quality management systems in teacher education, demonstrating how structured standards can enhance the consistency and effectiveness of training programs. Hartmann et al. provide a comprehensive review of professional learning communities, emphasizing the role of collaboration, shared goals, and continuous reflection in sustaining teacher development in digital STEM contexts.

The contributions gathered in this Research Topic demonstrate that digitalization in STEM education is not merely a matter of implementing new technologies; it requires aligning instructional design, teacher competencies, and professional development within coherent educational frameworks. Across the included studies, it becomes evident that the successful integration of digital tools depends on the interplay of well-structured learning environments, teachers' professional knowledge, their critical thinking, and their beliefs, in addition to supportive institutional conditions. In particular, the development of teachers' competencies, including TPACK-related knowledge, self-efficacy, and professional vision, emerges as a central factor for translating digital innovations into effective classroom practice. At the same time, the contributions highlight ongoing challenges, such as unequal access to high-quality professional development, variability in teachers' digital expertise, and the limited understanding of how motivational and emotional factors interact with digital learning environments across STEM domains. Future research should therefore focus on developing theory-driven approaches that integrate cognitive, motivational, and contextual perspectives and on strengthening collaborations between schools, universities, and out-of-school learning environments. These efforts are essential to ensuring that the digital transformation of STEM education is both sustainable and equitable. Taken together, the contributions to this Research Topic underscore that advancing digital STEM education requires a systemic and multi-layered perspective that integrates pedagogical innovation, teacher professional learning, and institutional support.
